# Utilizing effector-triggered immunity (ETI) as a robust priming agent to protect plants from pathogens

**DOI:** 10.1007/s44154-024-00204-7

**Published:** 2024-12-09

**Authors:** Faisal Islam, Muhammad Saad Shoaib Khan, Huan Chen, Jian Chen

**Affiliations:** 1 International Genome Center, Jiangsu University, Zhenjiang, 212013 China; 2 Joint Center for Single Cell Biology, School of Agriculture and Biology, Shanghai Jiao Tong University, 800 Dongchuan Road, Shanghai, 200240 China

Effector-triggered immunity is a complex and potent component of plant immunity and offers a strong defense mechanism against a broad spectrum of pathogens. ETI is initiated by direct or indirect recognition of specific pathogen effector proteins through plant intracellular immune receptors, known as Nucleotide-Binding Leucine-Rich Repeat Receptors (NLRs). This recognition leads to the induction of a wide range of immune responses, including the hypersensitive response (HR), a form of localized cell death that physically restricts the pathogen (Jones and Dangl 2006). ETI induces reactive oxygen species, antimicrobial compounds, and various signaling molecules (such as salicylic acid) that act further downstream in the amplification module to modulate immune responses (Islam et al. 2023).

Priming is a process through which a plant’s immune system is prepared for enhanced responses to future pathogen attacks. Primed plants can respond more rapidly and effectively to infections, often leading to systemic acquired resistance (SAR), where immune signals spread throughout the plant, effectively reducing disease incidence and improving overall resilience. The potential of ETI to act as a priming agent in the host plant against microbial pathogen infections could be a promising perspective for enhancing crop resilience and sustainability in agriculture (Lonjon et al. 2024). The mechanisms through which ETI can be used as a priming agent, its benefits, challenges of application, and broader implications on farming practices are discussed below, in the light of a recently published study by Lonjon et al. (2024).

## Mechanisms of enhanced plant defense via ETI priming: systemic and localized responses

**Fig. 1:**
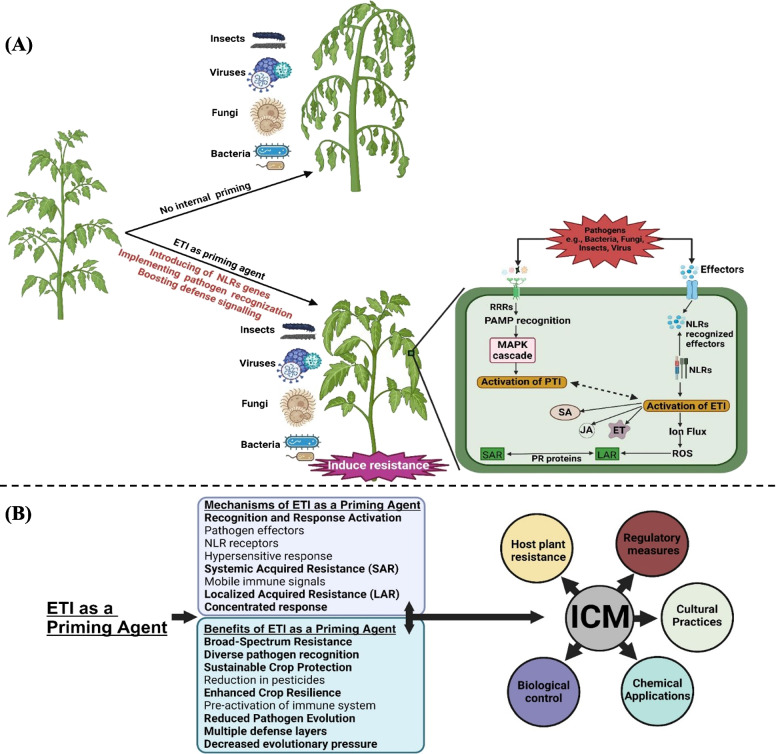
Schematic illustration of the effector-triggered immunity (ETI) mechanism in plants (**A**). Pathogen effectors are delivered into plant cells, recognized by NLR receptors, leading to immune activation, including hypersensitive response (HR), reactive oxygen species (ROS) production, antimicrobial compounds, and signaling molecules. ETI induces systemic acquired resistance (SAR) and localized acquired resistance (LAR), providing systemic and localized resistance. Simplified overview of utilizing ETI as a priming agent for plant protection in integrated crop management (ICM) program (**B**)

ETI can boost the plant immune system for stronger responses against subsequent infections through the process of systemic acquired resistance (SAR), in which mobile immune signals, upon induction, travel systemically across the plant and put it on alert to defend against a broad spectrum of pathogens (Fig. 1A). Important signaling molecules, including SA, pipecolic acid, and its derivative N-hydroxypipecolic acid, play a crucial role in establishing and maintaining the primed state in the context of SAR (Vlot et al. 2021). Apart from SAR, ETI can trigger localized acquired resistance (LAR), which concentrates the immune response in the area near the site of infection (Jacob et al. 2023). This spatial structure ensures that the main protective effect of ETI will be localized, effectively containing the pathogen’s growth while preventing its systemic spread (Jacob et al. 2023).

### Advantages of using ETI as a priming agent for plant disease resistance

The use of ETI as a priming agent could yield broad-spectrum resistance because it can recognize and respond to a wide range of pathogen effectors, which serves as a powerful strategy to protect plants against diverse pathogens such as bacteria, fungi, or viruses. For example, when non-virulent strains with ETI-electing effectors, such as HopBF1a or HopAB1n, were inoculated into tomato plants, the plants were able to activate their defense pathways in response to these effectors within 24–28 h (Lonjon et al. 2024). Consequently, when the virulent strain was introduced subsequently, its ability to cause disease was significantly suppressed. The study also found that when tomato plants were inoculated with non-virulent strain with ETI-electing effectors and the virulent strain simultaneously, disease symptoms were also abolished, showing the effectiveness of ETI-induced protection (Lonjon et al. 2024).

Using ETI as a priming agent will reduce environmental and health risks associated with pesticides use in agriculture; thus serving as a natural and sustainable alternative to chemicals-based pesticides. Integrating ETI-based biocontrol methods into pest management practices could lead to a more sustainable, ecofriendly agricultural system. Priming plants with ETI-eliciting effectors can enhance crop resilience by preparing the plant’s immune system for future attacks. This pre-activation of immunity in turn ensures an appropriate, rapid, and strong response to subsequent infection, thereby minimizing crop losses and stabilizing yield. Additionally, the implantation of ETI-based defense strategies could decrease evolutionary pressure on the pathogens to acquire or evolve resistance. Compared to single gene-mediated resistance, which is overcome easily by pathogens (Zhang et al. 2023), ETI implicates several layers of defense mechanisms that are relatively difficult for pathogen(s) to bypass or evade. However, it is important to note that many crop diseases are effectively managed through race-specific resistance strategies.

### Challenges in implementing ETI for enhanced plant immunity

The efficacy of ETI as a priming agent relies on the diversity and the specificity of expressed NLRs in a plant. For example, Lonjon et al. (2024) observed a higher degree of NLR gene conservation between cultivated and wild-relative tomato accessions. Some tomato varieties were resistant to all tested ETI effectors, while some are susceptible, showing significant immune-diversity in studied tomato varieties or accessions. A significant difference in ETI landscape of tomato and Arabidopsis was present against *Pseudomonas syringae*, indicating presence of distinct mechanisms for reorganization of pathogen effectors among studied plants (Lonjon et al. 2024). Similarly, some ETI responses in cultivated and wild tomato species are highly conserved, while some others are lost in certain wild species. For instance, HopAA and HopAR ETIs were absent in some wild tomato species. In other words, the study showed that various plant species with different NLR proteins could recognize the same effectors (Lonjon et al. 2024). Wild relatives could be used as donors of newly discovered NLRs, and breeding program should incorporate a broad range of NLRs to confirm protection against multiple pathogens.

The timing and dosage of effector application are of key considerations in realizing effective protection. Protection by ETI priming was found to be time-dependent, as it was found to be more effective when the ETI-inducing strain was pre-inoculated up to 48 h before the virulent strain inoculation (Lonjon et al. 2024). Such ETI effectors will likely require optimization of the application protocols or procedures to capture their full protective benefits. To investigate the potential finesse cost associated with ETI-induced protection in tomato plants, Lonjon et al. (2024) sprayed different concentration of *P. syringae* (PtoDC3000D36E, D36E::HopABn) (Wei et al., 2015) on tomato plants and found no significant growth deficit and change in fresh and dry weights of inoculated plants, validating no significant fitness cost (Lonjon et al. 2024). While the study indicated no growth deficit associated with ETI-induced protection (Lonjon et al. 2024), it is important to consider potential fitness costs in different plant species and environmental conditions. Continuous monitoring and evaluation are necessary to ensure that ETI-based priming does not adversely affect plant growth and productivity. Furthermore, ETI-based priming strategies needs a regulatory framework and may face adoption issues, which could be overcome by ensuring that they are safe, effective, and accessible to farmers.

### Integrating ETI insights into crop breeding and pest management

The knowledge of the ETI landscape and diversity of NLR genes offers opportunities for crop breeding programs to engineer disease-resistant crop species. Deployment of ETI-eliciting traits in commercial crops offers a long-term benefit to crop protection measures. ETI-based priming will be more effective if incorporated in holistic pest management which typically includes biological control, cultural practices, and reduced use of pesticides to improve overall crop health and resilience (Fig. 1B). More research should be conducted to extend the ETI landscape in other plant species and against a wider range of pathogens. In developing new biocontrol strategies, valuable information may lie in these evolutionary pressures that have shaped the ETI landscape. ETI-based priming of crop resilience would reduce diseases in crops, ensuring global food security in the face of climate change and reducing selection pressure on pathogens.

### Case study of leveraging ETI for improved disease resistance in tomato plants

Tomato is a vital food crop globally, and bacterial pathogens like *Pseudomonas syringae* could reduce its yield up to 75% in some regions (Panno et al. 2021; Seymour and Rose 2022). Lonjon and colleagues (2024) explored the ETI landscape in tomatoes to understand and improve tomato resistance against *P. syringae*. The primary objectives of the study were: i) to identify and characterize effectors that trigger strong ETI responses in tomato plants, and ii) to develop biocontrol methods that protect tomatoes from *P. syringae* infection using the identified ETI-elicitors. ETI screening was performed on five cultivated tomato varieties and two wild relatives, and an immunodiversity screening was conducted in 149 tomato lines. A high-throughput ETI screening was established using PsyTEC (*P. syringae* type III effector compendium) (Laflamme et al. 2020) to discover robust and reproduceable ETI responses. A quantitative test was conducted to investigate the extent to which ETI responses inhibited bacterial growth. Immunodiversity analysis was carried out via measuring variation in ETI responses across studied tomato cultivars and wild species.

The finding revealed that the ETI landscape appears more restricted in tomato than *Arabidopsis thaliana* (Dillon et al. 2019a and b, Lonjon et al. 2024)*.* Only HopAA1q, HopAR1h and the emergent effector HopBJ1b were significantly effective in inhibiting *P. syringae* growth during infection in tomato plants. The authors also identified six families of tomato effectors, including five previously uncharacterized effector families (HopAA, HopAR and HopBJ as class-I effectors; the other four are conserved classes) (Dillon et al. 2019a and b, Lonjon et al. 2024). Many ETI responses were conserved between cultivated and wild tomato species but in some cases, there were lost (especially in wild species). Some lines were resistant to all the tested ETI-elicitors, and others showed susceptibility, indicating significant immunodiversity among the 149 studied tomato genotypes. ETI-eliciting effectors protect tomatoes from *P. syringae* infection when delivered by a non-virulent strain, which suggest the potential of using ETI as a biocontrol strategy to protect tomatoes against *P. syringae*, offering a sustainable alternative to chemical pesticide applications (Lonjon et al. 2024).

## Conclusion

ETI priming seems a very promising and robust strategy to address pathogen-induced plant infections. By leveraging the natural immune responses of plants, ETI-based methods offer a sustainable, broad-spectrum, and effective crop protection tool. The study by Lonjon et al. (2024) provide valuable insight into the mechanisms, benefits, and challenges of ETI as a priming agent. Integrating ETI insights into crop breeding and pest management programs will lead to the development of new biocontrol methods to enhance resilience and sustainability in agriculture. Continued research and optimization are essential to fully realize ETI-based priming’s potential.

## Data Availability

Not applicable.
